# Broadening the scope: Multiple functional connectivity networks underlying threat conditioning and extinction

**DOI:** 10.1162/imag_a_00213

**Published:** 2024-07-03

**Authors:** Cody A. Cushing, Yujia Peng, Zachary Anderson, Katherine S. Young, Susan Y. Bookheimer, Richard E. Zinbarg, Robin Nusslock, Michelle G. Craske

**Affiliations:** Department of Psychology, University of California Los Angeles, Los Angeles, CA, United States; School of Psychological and Cognitive Sciences and Beijing Key Laboratory of Behavior and Mental Health, Peking University, Beijing, China; National Key Laboratory of General Artificial Intelligence, Beijing Institute for General Artificial Intelligence, Beijing, China; Institute for Artificial Intelligence, Peking University, Beijing, China; Department of Psychology, Northwestern University, Evanston, IL, United States; Social, Genetic and Development Psychiatry Centre, Institute of Psychiatry, Psychology and Neuroscience, King’s College, London, United Kingdom; NIHR Maudsley Biomedical Research Centre, King’s College London, London, United Kingdom; Department of Psychiatry and Biobehavioral Sciences, University of California Los Angeles, Los Angeles, CA, United States; Ahmanson-Lovelace Brain Mapping Center, Semel Institute for Neuroscience and Human Behavior, University of California Los Angeles, Los Angeles, CA, United States; Department of Psychiatry and Biobehavioral Sciences, David Geffen School of Medicine, Los Angeles, CA, United States; The Family Institute at Northwestern University, Evanston, IL, United States; Institute for Policy Research, Northwestern University, Evanston, IL, United States

**Keywords:** fMRI, threat, conditioning, extinction, functional connectivity

## Abstract

Threat learning processes are thought to be foundational to anxiety and fear-related disorders. However, the study of these processes in the human brain has largely focused on specific brain regions, owing partly to the ease of translating between these regions in human and nonhuman animals. Moving beyond analyzing focal regions of interest to whole-brain dynamics and connectivity during threat learning is essential for understanding the neuropathology of fear-related disorders in humans. In this study, 223 participants completed a 2-day Pavlovian threat conditioning paradigm while undergoing fMRI. Participants completed threat acquisition and extinction. Extinction recall was assessed 48 hours later. Using a data-driven group independent component analysis (ICA), we examined large-scale functional connectivity networks during each phase of threat learning. Connectivity networks were tested to see how they responded to conditioned stimuli during early and late phases of threat acquisition and extinction as well as during early trials of extinction recall. A network overlapping with the default mode network involving hippocampus, ventromedial prefrontal cortex (vmPFC), and posterior cingulate was implicated in threat acquisition and extinction. Another network overlapping with the salience network involving dorsal anterior cingulate cortex (dACC), mPFC, and inferior frontal gyrus was implicated both in threat acquisition and in extinction recall. Other networks overlapping with parts of the salience, somatomotor, visual, and frontoparietal networks were involved in the acquisition or in the extinction of learned threat responses. These findings help support the functional cooperation of specific brain regions during threat learning in a model-free fashion while introducing new findings of spatially independent functional connectivity networks during threat and safety learning. Rather than being a single process in a core network of regions, threat learning involves multiple brain networks operating in parallel performing different functions at different timescales. Understanding the nature and interplay of these dynamics will be critical for comprehensive understanding of the multiple processes that may be at play in the neuropathology of anxiety and fear-related disorders.

## 1 Introduction

Learning to respond to new threats needs to strike a balance between speed and accuracy to ensure survival and health in an environment. Environmental threats need to be readily identified in as little as one learning episode to ensure a potentially life-saving response. However, learning the probability of true danger from a potential threat requires multiple learning episodes to appropriately understand threat probabilities. Moreover, this learning needs to balance new associations with older long-term learning to ensure old learning is not too readily overwritten (Grossberg, 1987). Consequently, it is likely that multiple learning and memory systems are involved to balance these competing needs (McClelland et al., 1995). Deficits in threat learning processes can lead to behavioral outcomes resembling an anxiety disorder when these probabilities are not accurately learned (Abend et al., 2022;Britton et al., 2011;Robinson et al., 2013). Consequently, fear- and anxiety-related disorders are often thought to be characterized by aberrations in threat learning (Craske et al., 2017;Fenster et al., 2018;Zinbarg et al., 2022). For example, anxiety disorders have been associated with greater fear responses during threat acquisition leading to stronger threat associations, generalization of acquired threat responses to new stimuli, and impoverished threat extinction (Pittig et al., 2018). Anxiety is also thought to be related to how threat learning generalizes to new stimuli beyond the initial learning episode (Dunsmoor & Paz, 2015). As such, the Pavlovian threat learning paradigm has become a pillar in studies examining fear and anxiety-related processes due to its simplicity and utility in translational research from animal models to human participants.

Building from animal models, critical brain areas for threat learning have been identified such as the amygdala and hippocampus (Gray & McNaughton, 1996;Phillips & LeDoux, 1992). Functioning in these central regions is certainly informative for anxiety disorders as increased anxiety is associated both with increased threat responding and difficulty responding to ambiguous threats, as measured from amygdala activation (Im et al., 2017;Pittig et al., 2018). However, important regions such as the amygdala are often not detected as having a role in human fMRI studies of threat learning, suggesting a more diverse set of regions may be necessary to characterize threat learning processes in humans (Fullana et al., 2016;Visser et al., 2021;Young et al., 2021). Consequently, it has been necessary to expand beyond these critical nodes to the larger functional networks in which they operate. Broader univariate searches of the human brain have suggested a network of regions that respond to threat (i.e., respond greater to threatening conditioned stimuli [CS+] vs. safe conditioned stimuli [CS-]) including anterior insula, dorsal anterior cingulate cortex (dACC), dorsolateral prefrontal cortex (dlPFC), supplementary motor area (SMA), and ventral striatum (Fullana et al., 2016). Conversely, another set of regions seems to respond more to safety (i.e., respond greater to CS- vs. CS+) including ventromedial prefrontal cortex (vmPFC), posterior cingulate, and hippocampus (Fullana et al., 2016). Especially at the human level, it is likely that there are multiple circuits operating in parallel in what we would typically label “fear” learning (LeDoux & Pine, 2016). We use the term “threat learning” here—while noting that much of the literature traditionally refers to this paradigm as “fear learning”—as a means of capturing the multidimensional response to learned threat, which includes the subjective experience of fear as well as other physiological and cognitive responses that may or may not generate fear (Mobbs et al., 2009;Taschereau-Dumouchel, Michel, et al., 2022).

Much of the previous human neuroimaging work using the Pavlovian threat learning paradigm has used univariate contrasts to identify threat-sensitive brain regions (Fullana et al., 2016). However, functional connectivity is beginning to be used to understand the broader large-scale dynamics at play in human threat conditioning (Berg et al., 2021;Cisler et al., 2020;Marstaller et al., 2016,^2017^;Wen et al., 2021). Network analyses have become increasingly popular as a way to understand how distributed regions across the entire brain organize their activity in coordinated functions (Sporns, 2014). Understanding how large-scale brain networks operate during threat conditioning is likely to advance our understanding of the psychopathology of anxiety and fear-based disorders (Bressler & Menon, 2010;Menon, 2011). By understanding these large-scale dynamics, more effective neurobiologically targeted treatments may be developed by seeking to stabilize brain activity in these networks as a whole rather than trying to change complex symptomology by affecting singular brain regions in isolation.

Multiple large-scale connectivity networks (salience and central executive networks) have been shown to track overgeneralization of conditioned fear responses in post-traumatic stress disorder (Berg et al., 2021). Extinction of conditioned threat has also been shown to modulate functional connectivity widely across the brain in areas associated with default mode, frontoparietal, and ventral attention networks (Wen et al., 2021). These recent endeavors represent the need to look outside the canonical defensive threat/fear network regions to understand threat conditioning processes at a holistic whole-brain scale. Additionally, by examining connectivity, these recent studies are moving beyond just understanding which brain regions independently are involved in threat learning. They are exploring the important question of how brain regions coordinate with each other to form new memories about threat and fear and to integrate those new memories with the existing memory schemas already in place.

Here, we add to this growing body of research by utilizing data-driven group independent component analysis (ICA) methods on task-based fMRI data to investigate functional connectivity in brain networks involved in the acquisition and extinction of learned threat, as well as the recall of extinction memory after a 48-hour consolidation period. Group ICA has been established as a method for determining functional connectivity patterns in fMRI without having to rely on specific models of brain activity in both resting state and task-based data (Calhoun et al., 2001,^2009^;Igelstrom et al., 2015;Marstaller et al., 2016,^2017^;Webb et al., 2016). However, previous efforts examining group ICA functional connectivity in threat learning have been limited by small sample sizes (Marstaller et al., 2016,^2017^). By analyzing over 200 participants, this study represents one of the largest and most robust investigations of whole-brain dynamics during threat learning. Moreover, using group ICA, we are able to categorize multiple distinct, data-driven connectivity networks operating in parallel, allowing us to characterize different threat and safety sensitivities during learning.

Such methodology allows us to expand upon and provide additional insight into other recent explorations in whole-brain connectivity during threat learning, such as one that explored the temporal dynamics of fear learning and extinction at the single-trial level (Wen et al., 2021). This study was limited in its ability to isolate specific functional networks due to the nature of ROI-to-ROI pair-wise connectivity measures. The group ICA approach presented herein supplementsWen et al. (2021)by dividing whole-brain connectivity into spatially independent, temporally parallel networks and examining functional connectivity during threat conditioning within each one. Additionally, instead of relying upon models of predefined regions or anatomically restricted parcellations, the data-driven nature of group ICA inherently reveals which groups of brain regions are working in concert during threat and safety learning.

We predicted we would find multiple task-related functional connectivity networks during our 2-day threat learning task with this strategy. We expected to largely replicate the findings of other previous large-scale connectivity studies with independent connectivity networks overlapping or resembling the default mode, salience, and frontoparietal control networks (Berg et al., 2021;Marstaller et al., 2017;Wen et al., 2021) while also potentially finding novel results in additional networks due to the data-driven nature of our planned analysis. Importantly, our use of group ICA on the threat conditioning task data itself (as opposed to resting state data) will allow us to examine specifically which portions of these previously implicated canonical networks are engaged during the threat learning task. In addition, decomposing the BOLD signal into independent components enables us to characterize the conditioned stimulus response profiles of multiple functional connectivity networks parallel in time. Based on univariate findings (Fullana et al., 2016), we expect to see functional connectivity networks that respond more to threat (CS+) involving areas such as the insula, dACC, dlPFC, and somatomotor areas. Conversely, we expect to observe some functional connectivity networks responding more to safety (CS-) involving brain regions such as vmPFC, hippocampus, and posterior cingulate.

## 2 Methods

### 2.1 Participants

Participants were enrolled as part of a cross-site Brain, Motivation, and Personality Development (BrainMAPD) study at the University of California, Los Angeles (UCLA), and the Northwestern University (NU) that has been described in previous research (Anderson et al., 2023;Peng et al., 2023;Rosenberg et al., 2021;Young et al., 2021). We recruited 272 participants (mean age (s.d.) = 19.16 (0.52), 182 female). Participant recruitment focused on participants 18–19 years old and was structured to ensure an even distribution across threat and reward sensitivity, measured by the Behavioral Activation Scale (BAS) and Eysenck Personality Questionnaire-Neuroticism (EPQ-R-N) (Carver & White, 1994;Eysenck & Eysenck, 1993). Of 223 participants who underwent a SCID-5 interview as part of the larger study, 56 (21.53%) met criteria for an anxiety disorder with no comorbidity with depression, 18 (16.92%) met criteria for comorbid anxiety and depression, and 3 (1.15%) met criteria for depression with no comorbidity (Young et al., 2021). Prior reports on this BrainMAPD study sample have characterized and validated a tri-level symptom model resulting in 3 dimensional descriptors of participant symptomology in this sample: general distress, fears, and anhedonia-apprehension (Anderson et al., 2023;Naragon-Gainey et al., 2016;Peng et al., 2023;Rosenberg et al., 2021;Young et al., 2021). Therefore, analyses in the current paper include the 3 dimensional factors of general distress, fears, and anhedonia-apprehension as covariates to control for participant symptom levels in order to focus on typical threat learning processes irrespective of participant symptom level. Participants were ineligible if meeting any of the following criteria: left handedness, not fluent in English, history of traumatic brain injury, MRI contraindications, pregnancy, color blindness, lifetime psychotic symptoms, bipolar I disorder, substance use disorder in the past 6 months, or currently taking antipsychotic medication. Participants provided informed consent according to the procedures approved by UCLA and NU Institutional Review Boards. Participant retention was generally high as only 6 participants were excluded for failing to return for the second fMRI session with extinction recall. Participants were excluded from all fMRI analyses if they demonstrated excessive motion (defined as >10% outlier scans) in any of the 3 task phases (acquisition, extinction, and extinction recall). After exclusions for motion (62% of exclusions) and technical issues during data collection, 223 participants (152 female) were analyzed for acquisition and 208 participants were analyzed for extinction (140 female) and extinction recall (141 female).

### 2.2 Task

Participants completed a 2-day, three-phase (acquisition, extinction, and extinction recall) Pavlovian threat conditioning task as described previously (Milad et al., 2009;Peng et al., 2023;Rosenberg et al., 2021;Young et al., 2021) while undergoing an fMRI scan. Participants were instructed to focus on the fixation cross when it appeared and to pay attention to the stimuli presented as they will be asked questions about them later. They were also told they may or may not receive an electric shock during the task. At the end of each task phase, participants reported the contingency between each CS and the US. Participants were prompted to enter the “likelihood of receiving a shock if you saw this image again” on a 3-point scale. Skin conductance response (SCR) data were also collected during each task phase. Details of SCR recording can be found inSupplemental Methods. Acquisition and extinction occurred sequentially during the same scanning session while extinction recall was conducted in a separate fMRI session at least 48 hours later (mean days apart (s.d.) = 2.76 (2.48)). Each trial began with 3 seconds of a context image presentation (office or conference room setting with noncolored lamp) followed by 6 seconds of CS presentation (lamp color changing to red, yellow, or blue). During acquisition, the unconditioned stimulus (US) was delivered via electric shock that coterminated with the CS+. Stimulus offset was followed by a varied intertrial interval of 12 to 18 seconds (mean 15 seconds). Acquisition occurred with one context image while extinction and extinction recall occurred with the other context image. Context image and CS lamp-color assignments were counterbalanced across participants. Acquisition consisted of 8 trials of a CS+ that became the extinguished CS (CS+E), 8 trials of a CS+ that would remain unextinguished (CS+U), and 16 trials of a CS- that was never paired with shock. CS+ stimuli were reinforced with shock on 5 out of 8 trials each (62.5% reinforcement rate). Extinction contained 16 CS+E trials and 16 CS- trials. Extinction recall contained 8 CS+E trials, 8 CS+U trials, and 16 CS- trials. No shocks were delivered in either the extinction or extinction recall phases. The US was an electric shock consisting of 10 1-ms pulses at 20 Hz lasting 500 ms. Shocks were delivered to the left bicep with a DS7a constant current high-voltage stimulator (Digitimer Ltd, England) at UCLA and using a STMISOC constant voltage stimulator (Biopac Systems Inc., USA) at Northwestern University. US intensity was determined via a work-up procedure before fMRI scanning to elicit a sensation that was “uncomfortable but not painful” and “took some effort to tolerate,” but at a level they would be willing to tolerate for the duration of the experiment. Delivery of the US was balanced throughout the acquisition phase such that early and late trials contained the same number of reinforced trials.

### 2.3 MRI acquisition parameters

MRI data were collected using a PRISMA 3T scanner with a 64-channel head coil in a cross-site study at the UCLA Ahmanson-Lovelace Brain Mapping Center and the Northwestern University Center for Translational Imaging. Acquisition parameters were identical at both sites. High-resolution T1-weighted structural images were collected using a magnetized prepared rapid acquisition gradient echo (MPRAGE) sequence (voxel size = 0.8 mm^3^, TR/TE/flip angle = 2300 ms/2.99 ms/7^o^, FOV = 256 mm^2^, 208 slices). Functional images were acquired parallel to the AC-PC line with a multiband sequence (voxel size = 2.0 mm^3^, TR/TE/flip angle = 2000 ms/25 ms/80^o^, FOV = 208 mm^2^, 64 slices, multiband acceleration factor = 2) collecting 380 volumes for each task phase.

### 2.4 MRI preprocessing

Structural T1 images were intensity normalized and brain extracted using optiBET (Lutkenhoff et al., 2014). The brain extracted T1 image was then segmented into white matter, gray matter, and cerebrospinal fluid using FAST (Y. Zhang et al., 2001).

Functional images were preprocessed separately for the acquisition, extinction, and extinction recall runs. Motion outliers were calculated for functional images using*fsl_motion_outliers*before any preprocessing took place. Then, functional images were motion corrected, smoothed with a 4 mm FWHM kernel, and nonlinearly registered into the MNI152NLin6Asym standard space using 12 degrees of freedom with FSL’s FEAT (FMRIB’s Software Library,www.fmrib.ox.ac.uk/fsl). Motion components were then automatically detected and removed using ICA-AROMA (Pruim et al., 2015). ICA-AROMA was used as it has been found to be one of the most effective methods of removing motion from fMRI data without discarding large amounts of data (Parkes et al., 2018). Following ICA-AROMA, data had linear and quadratic trends removed along with 6 head motion parameters from FSL and white matter and cerebrospinal fluid time courses using AFNI (Cox, 1996;Cox & Hyde, 1997). A high-pass filter was also applied to remove frequencies below 0.008 Hz during this step to remove potential sources of noise. A low-pass filter was not applied to prevent the filtering of any task-relevant content contained in the high frequencies. Finally functional data were transformed into the standard space using parameters from the FSL nonlinear registration.

### 2.5 Group ICA connectivity and dual regression

Similar to preprocessing, a separate group ICA connectivity analysis was performed for the acquisition, extinction, and extinction recall fMRI runs to reveal data-driven functional connectivity networks. Preprocessed functional data for all participants were submitted to group ICA in Melodic from FSL using the temporal concatenation method. Group ICAs were limited to 20 independent components (ICs) based on previous work (Webb et al., 2016) and to limit the number of multiple comparisons. The group ICA was masked to brain tissue only using the FSL standard brain mask. The result of each group ICA is 20 ICs consisting of spatial maps (functional connectivity networks) and time courses of activity within these connectivity networks. For a particular IC, the spatial map indicates which brain regions specifically are exhibiting functional connectivity during the scan. The corresponding IC time course indicates the activation of a functional connectivity network at a given time point. Group IC maps were thresholded at Z = 4 for generation of figures and identification of key network regions. Any components that resembled physiological noise or did not have significant contributions from the cortex (e.g., cerebellar networks) were discarded from further analysis. This resulted in 16 functional connectivity networks analyzed during each task phase. Dual regression was also performed using FSL to obtain participant-specific spatial maps and time courses for each IC to enable statistical analysis. As this group ICA combined data from two scanning sites, we also performed analyses testing for any potential site differences in the estimated functional connectivity networks as described inSupplemental Methods.

### 2.6 Modeling functional connectivity network response to task conditions

To find how functional connectivity networks responded to the conditions of the task, a GLM was fitted to each participant-specific time course for each IC using pyMVPA in python (Hanke et al., 2009). This process is identical to modeling a typical univariate whole-brain GLM, but rather than a voxel time course being used as a dependent variable, the time course of an IC (functional connectivity network) is used. Specifically, the conditions of CS+ and CS- were modeled separately for the early and late phases of each run for acquisition and extinction. During acquisition, the CS+ condition consisted of both CS+E and CS+U stimuli pooled together. The unconditioned stimulus (US) was also modeled during acquisition. For extinction recall, early and late unextinguished CS+ (CSU), extinguished CS+ (CSE), and CS- were modeled. Early and late represented the first and last four trials of each stimulus type, respectively. A regressor was also included for context presentation (office or conference room image) during each experimental phase. Motion outliers identified in preprocessing were included as additional nuisance regressors in the GLM to minimize effects of movement on parameter estimation. GLMs were modeled with an SPM-style hemodynamic response function model including a temporal derivative to account for temporal differences in slice acquisition. As this was an event-related design not requiring participant responses, all regressors were specified with a duration of 0 seconds at the onset of each stimulus for an event-related response over epoch response in order to minimize influence of temporally adjacent events (e.g., stimulus offset, US presentation, etc.) and account for rapidly habituating visual responses to stimuli.

### 2.7 Statistical analysis

To identify which functional connectivity networks were sensitive to task conditions, a mixed linear model was calculated for each IC within each task phase (acquisition, extinction, and extinction recall) using the statsmodels package in python. In acquisition and extinction, the dependent variable of IC Beta estimate was tested for the interaction of fixed effects condition (CS+/CS-) and time (early/late) for each IC. Due to most CS+ trials in acquisition being followed by a US (reinforcement rate 62.5%), we did not exclude reinforced CS+ trials in our main analysis to preserve statistical power. As a supplementary analysis (described inSupplemental Methods), we examined unreinforced trials only to test for potential influences of the US (seeSupplemental Results). For extinction recall, we were only interested in the early phase of the task so a model of fixed effect of condition (early CSU vs. early CSE) was tested. As some participants potentially had symptoms associated with mood or anxiety disorders (described above), additional covariates were included to control for the effect of these symptoms on threat learning. The three factors of general distress, fears, and anhedonia-apprehension were included as fixed effects to control for participant symptom levels. We also explored the relationship between these participant symptoms and functional connectivity network responses inSupplemental Results. Additionally, participant medication status was included as covariate (fixed effect) to account for any influence psychotropic medications may have on threat learning processes. Participant and scan site (UCLA/NU) were included as random effects with participant nested within scan site for each model. Scan site’s inclusion in the model should account for any variance explained by cross-site differences, but a cross-site analysis was performed to assess replication of our reported effects at each study site (described inSupplemental Methods). With a model for each IC not discarded for resembling noise/noncortical sources, 16 total models were tested for each task phase. Results of each model were Bonferroni corrected for multiple comparisons.

### 2.8 Canonical network overlap

In order to characterize with which canonical networks each IC network identified in our analyses overlapped, we calculated the overlap between our IC spatial maps and seven canonical networks (Schaefer et al., 2018): visual, dorsal attention, control, default mode, limbic, salience/ventral attention, and somatomotor. Canonical network masks were generated by combining and binarizing individual parcels belonging to each of the 7 networks from the 100 parcel 7 network Schaefer atlas (Schaefer et al., 2018). Thresholded IC spatial maps (described above) were binarized. Overlapping voxels between the thresholded IC maps and each network mask were determined by multiplying the two binarized images together. Then, the percentage of overlap between each IC network and each canonical network was calculated by dividing the number of overlapping voxels by the total number of voxels in each canonical network mask.

## 3 Results

### 3.1 Assessment of threat learning

This task was successful in inducing threat acquisition and extinction as assessed with behavioral contingency ratings collected at the end of each task run (previously reported inYoung et al., 2021). SeeSupplemental Resultsfor participant contingency ratings and SCR from each task phase. To explore the relationship between the responses of our functional connectivity networks and behavioral contingency ratings as well as SCR, we performed Pearson correlations between our reported neural findings and behavioral/SCR measures (seeSupplemental Results).

### 3.2 Stable network across acquisition and extinction

**Fig. 1.**
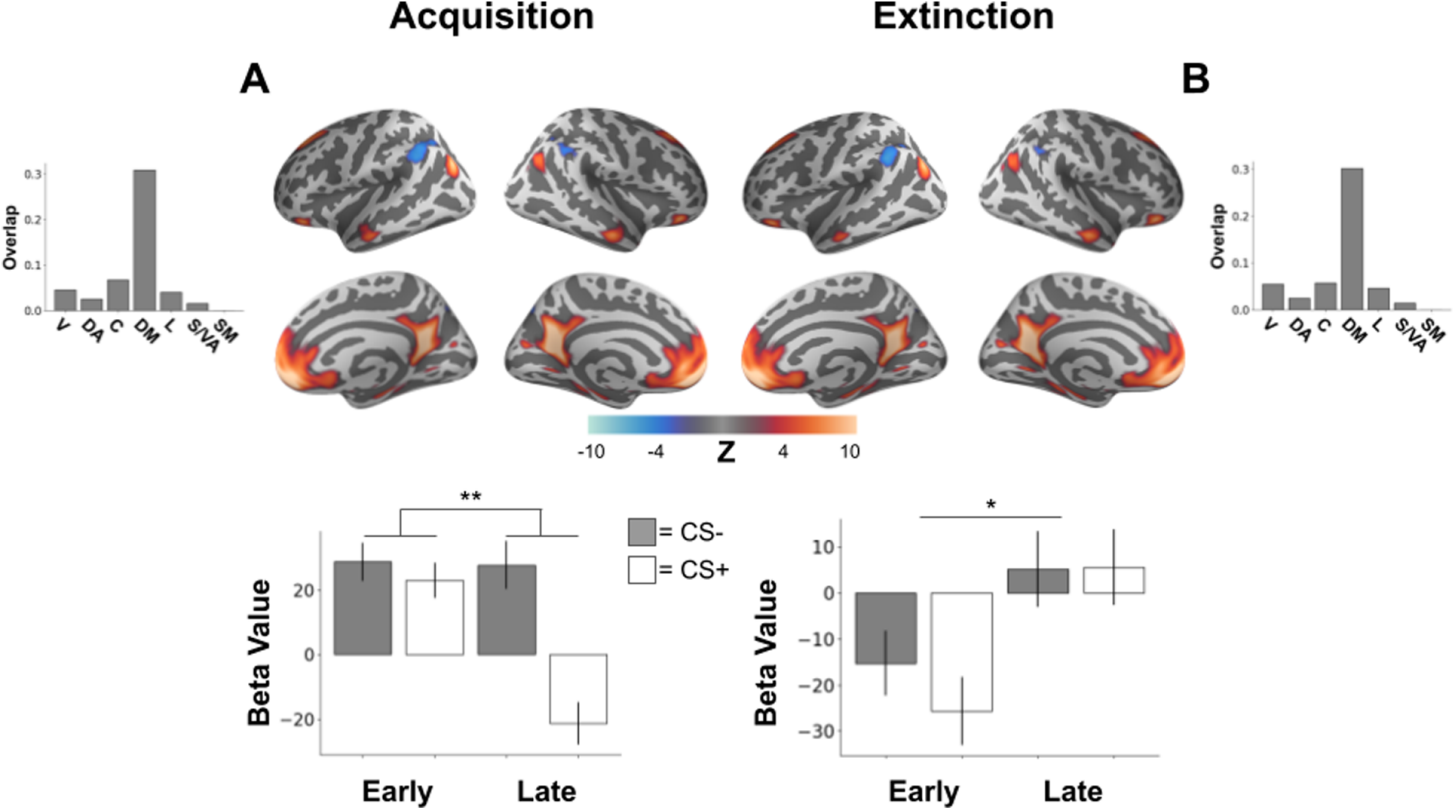
Brain network demonstrating acquisition and extinction of learned threat. Brain plots show thresholded independent component (IC) spatial maps. Bottom bar plots show IC-specific GLM parameter estimates. Top bar plots show connectivity network overlap with canonical networks. (A) A distributed brain network involving bilateral hippocampus, vmPFC, and posterior cingulate demonstrated a significant interaction between CS-type (CS-/CS+) and time (early/late) during threat acquisition. During late acquisition, the connectivity network demonstrated increased activity to the CS- compared with the CS+. (B) This same brain network involving bilateral hippocampus, vmPFC, and posterior cingulate was observed during threat extinction with activity in the connectivity network increasing from early to late extinction. *p < 0.05, **p < 0.01, V = visual, DA = dorsal attention, C = control, DM = default mode, L = limbic, S/VA = salience/ventral attention, SM = somatomotor.

During threat acquisition, there was a significant interaction between CS-type and time (*F*(1,883) = 15.93,*p*= 0.0011 Bonferroni corrected) in a connectivity network including vmPFC, OFC, hippocampus, angular gyrus, posterior cingulate, and retrosplenial cortex overlapping with the default mode network (Fig. 1A). This interaction was characterized by no initial difference in responding between CS+ and CS- during early trials (*t*(222) = 0.99,*p*= 0.32), while in late trials, connectivity network activity was significantly greater for CS- than for CS+ (*t*(222) = 6.71,*p *< 0.001). Such late decreases in response to CS+ are thought to identify regions important for threat extinction (Garcia et al., 1999; Hennings et al., 2020; Phelps et al., 2004).

During extinction, we observed a main effect of time in the same network involving vmPFC, hippocampus, and posterior cingulate from threat acquisition (*F*(1,823) = 11.42,*p*= 0.012 Bonferroni corrected,Fig. 1B). This network’s spatial pattern strongly correlated with the network shown inFigure 1Afound during acquisition (Pearson’s*r*= 0.98), supporting consideration of this as the same functional connectivity network observed during acquisition. This connectivity network, overlapping with default mode network, demonstrated decreased activation in response to CS stimuli during early extinction that increased to positive activation during late extinction. This change in response likely tracked extinction learning due to the positive increase over the extinction period as opposed to a decrease over the extinction period as might be expected from habituation or unrelated processes.

Interestingly, there was no CS-type sensitivity during threat extinction despite immediately following threat acquisition. No IC networks demonstrated interactions of CS type and time or main effects of CS type. This may be due to a dishabituation effect for the CS- due to the break between fMRI scans following acquisition and before extinction. However, potential extinction processes could be tracked through observed main effects of time.

### 3.3 Stable network across acquisition and extinction recall

**Fig. 2.**
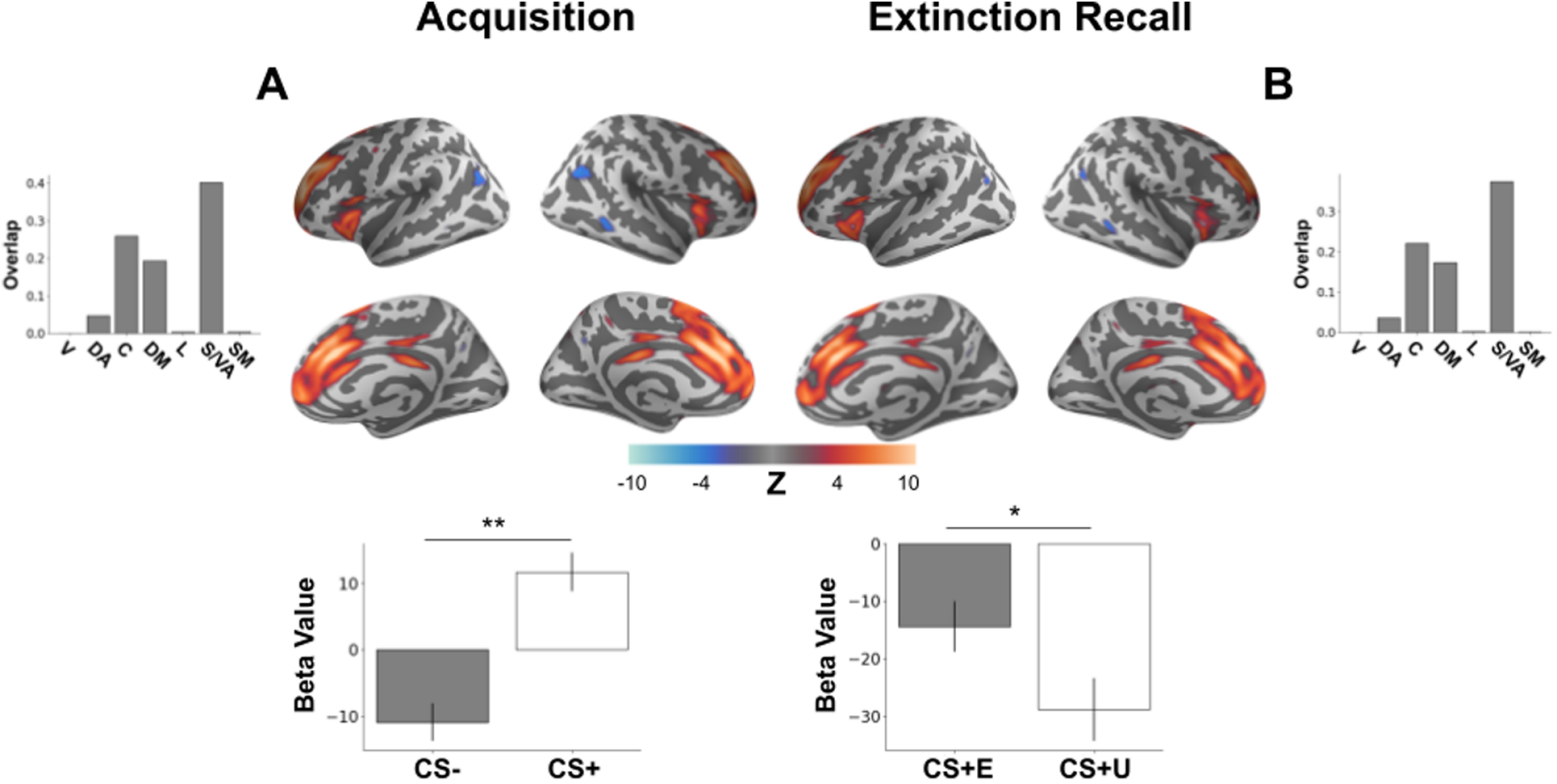
Brain network demonstrating acquisition of learned threat and recall of extinction memory. Brain plots show thresholded independent component (IC) spatial maps. Bottom bar plots show IC-specific GLM parameter estimates. (A) A distributed brain network consisting of dorsal anterior cingulate cortex (dACC), mPFC, and inferior frontal gyrus demonstrated an effect of CS type during threat acquisition with greater connectivity network activation elicited by the CS+ than by the CS-. (B) This same brain network was observed during extinction recall with significantly decreased connectivity network activation elicited by the unextinguished CS+ (CS+U) compared with the extinguished CS+ (CS+E). *p < 0.05, **p < 0.01, V = visual, DA = dorsal attention, C = control, DM = default mode, L = limbic, S/VA = salience/ventral attention, SM = somatomotor.

During acquisition, a network overlapping most with the salience/ventral attention network including dorsal anterior cingulate cortex (dACC), mPFC, and inferior frontal gyrus exhibited a main effect of CS type (*F*(1,883) = 15.70,*p*= 0.0013 Bonferroni corrected,Fig. 2A). Specifically, activation in the network was greater for the CS+ than for the CS-.

As only the early period of extinction recall was of experimental interest, we ran a model examining only the main effect of CS type in the early phase of the task (early CS+E vs. early CS+U). This revealed the same connectivity network found during threat acquisition that exhibited a greater decrease in activation (*F*(1,407) = 10.15,*p*= 0.025 Bonferroni corrected) for the unextinguished CS+ (CS+U) than for the extinguished CS+ (CS+E,Fig. 2B). This network’s spatial pattern strongly correlated with the network fromFigure 2Afrom acquisition (Pearson’s*r*= 0.97) supporting consideration of this as the same connectivity network observed during acquisition. These results showcase the importance of this network both in the acquisition of threat learning and in the expression of extinction memory. For the sake of completeness, we ran the same analyses on the late period of extinction recall. No significant differences were found in any network (all*p *> 0.05), making the previous effect unique to the early part of extinction recall likely due to the CS+U undergoing extinction during the recall period.

### 3.4 Individual networks during acquisition

**Fig. 3.**
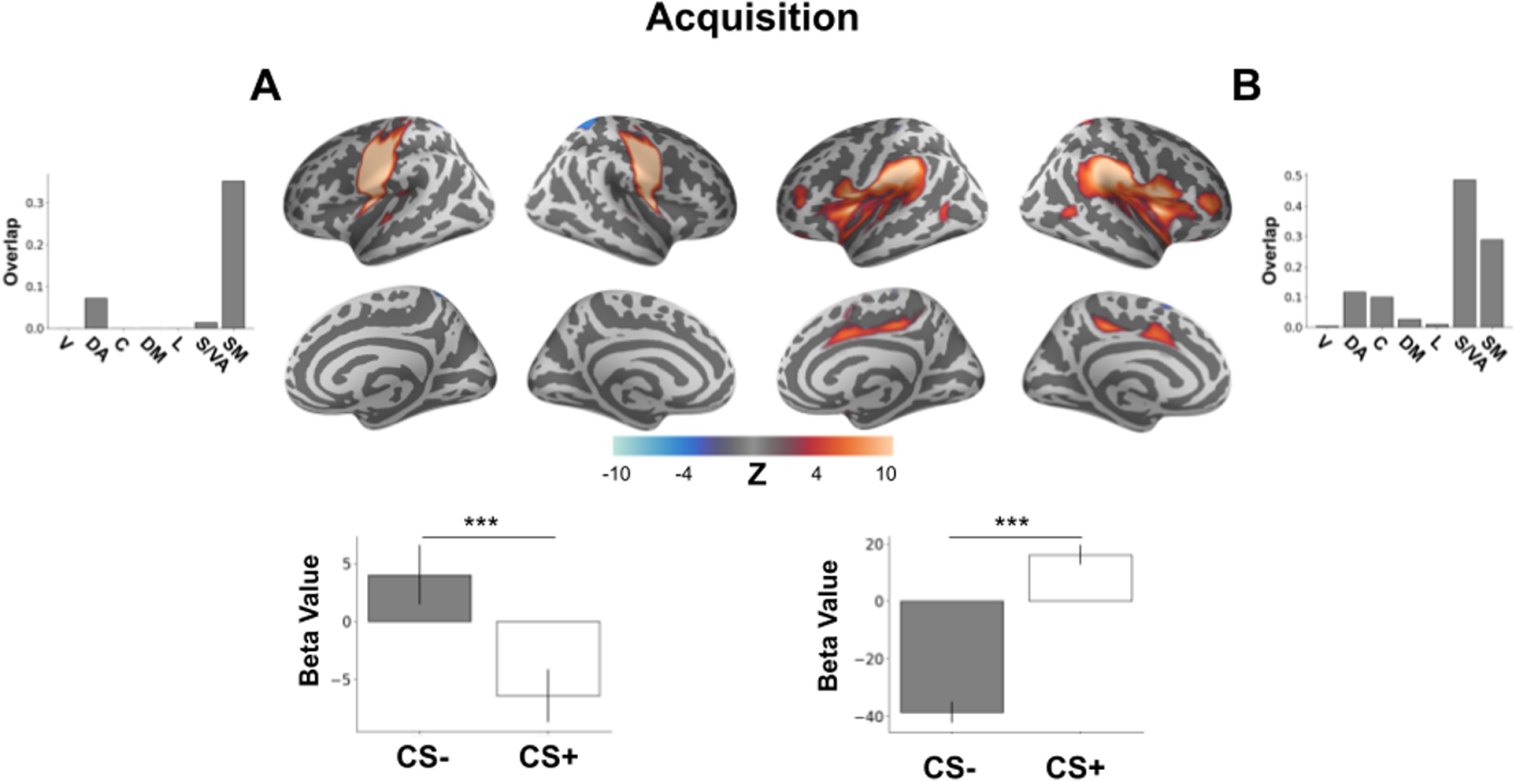
Brain networks involved in learned threat acquisition. Brain plots show thresholded independent component (IC) spatial maps. Bottom bar plots show IC-specific GLM parameter estimates. Top bar plots show connectivity network overlap with canonical networks. (A) A brain network involving precentral and postcentral gyri as well as the left insular cortex demonstrated increased activity elicited by the CS- and decreased activity elicited by the CS+ during threat acquisition. (B) A brain network involving the insula, middle frontal gyrus, cingulate gyrus, and OFC. This network demonstrated acquired threat response with increased activity induced by the CS+ and decreased activity elicited by the CS-. ***p < 0.001, V = visual, DA = dorsal attention, C = control, DM = default mode, L = limbic, S/VA = salience/ventral attention, SM = somatomotor.

We identified two additional functional connectivity networks involved in acquisition with opposing stimulus-response profiles. One connectivity network demonstrated a main effect of CS type with CS- responding being significantly greater than CS+ responding (*F*(1,883) = 14.13,*p*= 0.003 Bonferroni corrected). The network consisted of the precentral and postcentral gyri and insular cortex, overlapping with the somatomotor network (Fig. 3A). The other network spanned the entirety of insular cortex including anterior insula along with cingulate gyrus, inferior frontal gyrus, and OFC (Fig. 3B). This network also demonstrated a main effect of CS type (*F*(1,883) = 109.91,*p *< 0.001 Bonferroni corrected,Fig. 3B), but with activation to the CS+ being significantly greater than activation to the CS-.

### 3.5 Individual networks during extinction

**Fig. 4.**
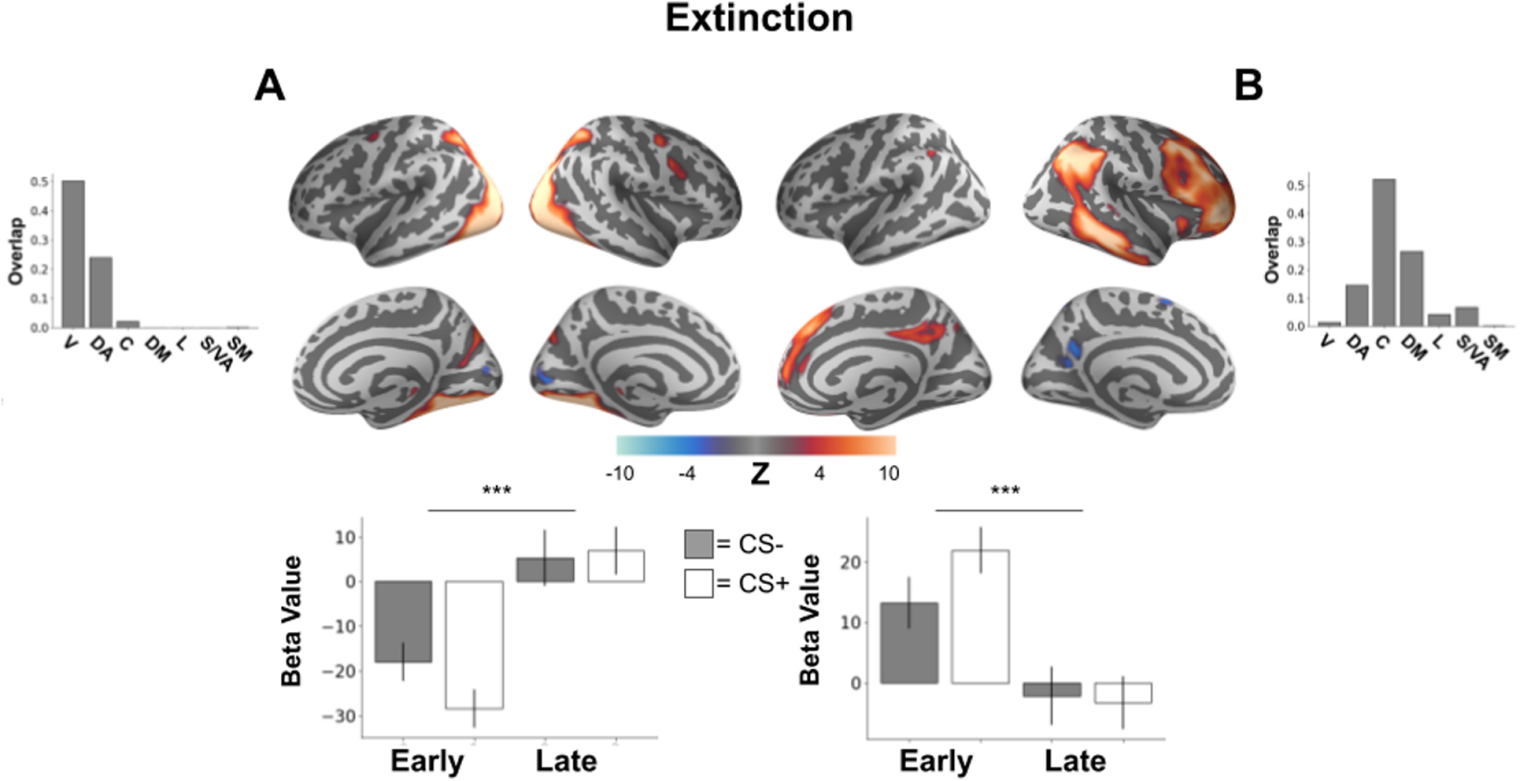
Brain networks involved in the extinction of learned threat response. Brain plots show thresholded independent component (IC) spatial maps. Bottom bar plots show IC-specific GLM parameter estimates. Top bar plots show connectivity network overlap with canonical networks. (A) A brain network involving lateral occipital cortex as well as fusiform cortex demonstrates a significant increase in connectivity network activity in response to CS stimuli from early to late trials in the extinction learning phase. (B) A brain network involving angular gyrus, frontal gyrus, and posterior cingulate showed significantly reduced connectivity network activity in response to CS stimuli from early to late trials in the extinction learning phase. ***p< 0.001, V = visual, DA = dorsal attention, C = control, DM = default mode, L = limbic, S/VA = salience/ventral attention, SM = somatomotor.

A network, overlapping with the visual network involving lateral occipital cortex and fusiform cortex, showed a main effect of time with an increase in activation from negative to positive over the extinction period (*F*(1,823) = 30.18,*p *< 0.001 Bonferroni corrected,Fig. 4A). Though this network was not observed during the threat acquisition process, this network activity also potentially tracks extinction learning due to the increase of responding over the task period.

Finally, we observed a main effect of time in another connectivity network, involving angular gyrus, frontal gyrus, and posterior cingulate, overlapping most with the control network (*F*(1,823) = 21.49,*p *< 0.001 Bonferroni corrected,Fig. 4B). However, as the activation of this network decreased from early extinction to late extinction, it is difficult to attribute this result to extinction learning directly without stimulus-specific evidence as it could also represent stimulus habituation or other unrelated processes.

## 4 Discussion

In this study, we examined functional connectivity networks during a 2-day Pavlovian threat learning paradigm using fMRI. With a data-driven group independent component analysis, we compared how spatially independent functional connectivity networks responded to conditioned stimuli during acquisition and extinction of threat responses as well as extinction recall a full 48 hours later. This revealed multiple distinct brain networks involved in the acquisition, extinction, and recall of extinction memory for learned threat. A network persisting across task phases, overlapping with the default mode network involving hippocampus, vmPFC, and posterior cingulate, was involved in both the acquisition and extinction of learned threat. An additional persisting network, overlapping with the salience network involving dACC, mPFC, and inferior frontal gyrus, was involved in the acquisition of learned threat as well as the expression of extinction memory during extinction recall. Additional networks overlapping with parts of the salience/ventral-attention and somatomotor networks were involved in the acquisition of learned threat responses. Portions of the visual and frontoparietal control networks were implicated in threat extinction.

The finding of a network involving hippocampus and vmPFC is consistent with previous work that has focused on these regions as part of a network that consistently responds to threat conditioning paradigms (Giustino & Maren, 2015;Picó-Pérez et al., 2019). It is worth noting that these regions were found to be functionally connected in the current work without the imposition of a model through the a priori selection of regions of interest. This strengthens the argument for them as canonical threat learning regions while demonstrating the coordination of these regions in a self-contained functional connectivity network. Responses specific to the conditioned cue developed late in the threat acquisition phase, indicating this network’s involvement in the learning aspect of threat acquisition. Activation in this functional connectivity network then increased during the extinction process, again suggesting a learning process over the extinction period.

As the observed network partially overlaps with the canonical default mode network, this adds to a building body of evidence in human neuroimaging implicating the default mode network in threat learning (Berg et al., 2021;Marstaller et al., 2017;Wen et al., 2021;Zidda et al., 2018). While the default mode network itself has often been associated with general “deactivation” in response to task demands (Raichle et al., 2001), it should be noted this network decreased it’s activity specifically to the CS+, but not the CS- during threat acquisition indicating an active role in threat learning rather than a generic task-related decrease in default mode activity. Moreover, activation increased in this network during extinction in contrast to what was observed during acquisition. Taken together, these results suggest an active role in threat learning for this portion of the default mode network beyond a general decrease in activation according to task demands. Due to the proposed role of threat learning in fear-based disorders, this supports the default mode network as a potential hub in the pathophysiology of anxiety and fear-based disorders (Marstaller et al., 2017;Menon, 2011;Son et al., 2023;J. Zhang et al., 2023;Zidda et al., 2018). As this network was involved in threat conditioning and extinction in the current study, it is a prime candidate for future studies concerning the relation of this network to the pathophysiology of anxiety disorders and fear-related disorders.

We also observed a stable network across threat acquisition and extinction recall in dACC, inferior frontal gyrus, and mPFC overlapping partly with the salience network. As the observed stimulus specificity during acquisition in this network was not specific to the late acquisition period, it is possible that this network detects learned threats at a rapid rate (e.g., one-trial learning). This network was also the only network to show conditioned stimulus specificity (between the extinguished and unextinguished conditioned stimuli) following the acquisition period. As this stimulus specificity was observed 48 hours after acquisition and extinction, it is implicated in long-term storage of learned threat responses. This underscores the importance of this network of regions in the threat learning process as a site of potentially both rapid and lasting threat memory acquisition. These results may be of broad clinical significance in understanding anxiety and fear-related disorders as the salience network itself has been found to track fear generalization and symptom severity in post-traumatic stress disorder (Berg et al., 2021). Perhaps a rapid learning rate within this network predisposes it to overgeneralized and persistent threat responses.

Potential rapid threat learning within the salience network is further supported by the current finding of an additional insula-centered connectivity network during threat acquisition where conditioned stimulus specificity (i.e., greater activation in response to CS+ compared with CS-) emerged early. Both networks overlapping most with the salience/ventral attention network demonstrated the same stimulus-response profile (CS+ > CS-), supporting the salience network’s role in learned threat detection. The salience network involving the insula is thought to be responsible for detecting salient events in a bottom-up fashion while coordinating other networks to access attention, working memory, and motor systems in response to salient events (Uddin, 2015). Consequently, the salience network demonstrating threat learning early in the acquisition phase supports this model of function, as the CS+ stimulus should be salient following the first delivery of the US. Due to the insula and salience network’s role in bottom-up processing (Menon & Uddin, 2010;Uddin, 2015), this may indicate greater bottom-up processing for the CS+ in these networks during threat acquisition.

In addition, a functional connectivity network overlapping primarily with the somatomotor network involving pre- and postcentral gyri was involved in threat acquisition. As these regions correspond to the motor and somatosensory cortices, connectivity in this network may reflect the preparation of motor responses to threat. Important to note, activation in this functional connectivity network was greater for the safety stimulus, CS-, compared with the threat stimuli, CS+, in a response profile similar to the network overlapping with the default mode network. A recent meta-analysis similarly found greater activation in the primary somatosensory cortex to the CS- versus the CS+ (Fullana et al., 2016). So, this finding may represent a safety signal, relating to approach-oriented motor behaviors. Future research directly examining approach-avoidance responses to stimuli will be needed to assess this possibility.

During threat extinction, beyond the network overlapping with the default mode network, we observed two functional connectivity networks overlapping with the visual and frontoparietal control networks. Functional connectivity in the visual network involving lateral occipital and fusiform cortices demonstrated an increase in activation during extinction from early to late trials. This increase in network activity may represent processing of visual contextual information to help process the new safe context as well as the CS+’s transition itself to no longer signaling threat. Conversely, activation in this frontoparietal control connectivity network decreased from early to late trials during extinction. As the frontoparietal network is associated with top-down regulation and task flexibility (Cole et al., 2013;Dosenbach et al., 2006;Power et al., 2011;Rossi et al., 2009), this decrease in activation may represent a reduction in top-down regulation or attentional processes that are necessary during early extinction when it is uncertain for participants whether stimuli may still represent a threat. However, as this was a general decrease in activation across both stimulus types, this decrease may simply reflect stimulus habituation or other unrelated processes.

Interestingly, we did not observe conditioned stimulus specificity during the extinction period in any examined network. This is peculiar given the strong awareness participants had of threat contingencies (reported inYoung et al., 2021). A lack of stimulus specificity is all the more perplexing given the extinction phase immediately followed the threat acquisition phase, in which conditioned stimulus specificity was widely observed. Stimulus specificity was again observed at the beginning of extinction recall. Nonetheless, the result broadly matches other whole-brain connectivity findings using a similar paradigm (Wen et al., 2021) in which no conditioned stimulus-specific response was observed early in extinction (though, unlike our study, they did observe stimulus specificity late in extinction). One potential explanation is a dishabituation effect for the CS- due to the inherent break (e.g., 1–2 minutes) between fMRI scans following acquisition and before extinction (Zbozinek et al., 2022).

Strengths of our study include a large sample size of more than 200 participants. As neuroimaging frequently suffers from low sensitivity and underpowered sample sizes (Thirion et al., 2007), the findings here can be considered more robust than many other neuroimaging studies. Despite the size of this sample, significant amygdala involvement was conspicuously missing from any of the networks found in this current analysis. This matches large meta-analyses as well as other findings that find a minimal role (if any) for the amygdala in human fear conditioning paradigms (Fullana et al., 2016;Visser et al., 2021;Young et al., 2021). The lack of significant activity in the amygdala during threat learning paradigms in human neuroimaging has been attributed to less salient threat in human studies compared with animal studies, as well as a general difficulty in measuring transient responses with fMRI (Fullana et al., 2016,^2019^;Somerville et al., 2013). Moreover, as we have argued above, emotions evoked by threat versus safety may rely upon distributed networks rather than single regions such as the amygdala. On the other hand, with enough statistical power, reliable contributions to threat conditioning and safety learning can be observed in the human amygdala with fMRI when analyzed specifically (Wen et al., 2022).

The current study is not without limitations, however. We recognize that the totality of these results across many networks does not necessarily congeal into a singular cohesive narrative. By using a data-driven method like group ICA with minimal network inclusion criteria, we are reporting results that may not immediately fit into canonical theories or thinking about threat learning. As a result, the findings independently validate the recent ideas that the default mode and salience networks are important for human threat conditioning in a model-free fashion (Berg et al., 2021;Marstaller et al., 2017,^2021^). Relatedly, there are opportunities to test more nuanced hypotheses in the current study compared with what has been done in the past. However, future studies building on this work will be better equipped to investigate more specific hypotheses. We view the current study as a stepping stone to more nuanced investigations by providing a solid and well-powered foundation to a field in the early stages of such large-scale investigations. Consequently, this collection of results may prove important for expanding the field beyond the traditional fear network regions, pending further replications and extensions relating brain networks to behavioral responses and symptom dimensions. Multiple circuits underlie the complex of physiological, behavioral, and subjective responses and their coordination for an adaptive or coherent response proportional to the level of threat imminence (Bolles & Fanselow, 1980;Mobbs et al., 2007). As these processes may potentially become dissociated during certain treatments for anxiety disorders based on threat learning principles—for example, amygdala and skin conductance response reduction with no corresponding reduction in self-reported fear (Cushing, Lau, et al., 2023;Taschereau-Dumouchel et al., 2018)—it is important to understand the neural circuitry behind each of these processes to most effectively tailor future treatments (Cushing, Dawes, et al., 2023;Taschereau-Dumouchel, Cushing, et al., 2022;Varkevisser et al., 2023).

Additionally, this cross-sectional dataset is limited in the conclusions that can be drawn. Participants analyzed in this study were relatively young (average age of 19 years) with low variance in age due to these data coming from a larger, longitudinal study looking at developmental changes from late adolescence into early adulthood. In the future, a longitudinal analysis of functional connectivity during threat learning and extinction will inform the stability of these connectivity networks over time and how their function relates to development of symptoms around anxiety and fear-based disorders. The results of this study also do not speak of the subjective experience of fear itself, as participants were not asked to rate their fear levels during or after the task. Consequently, the results of this study should be interpreted in the lens of general threat responses encompassing both nonconscious physiological threat responses and consciously experienced fear and threat awareness. Additionally, despite our large sample size, our data-driven group ICAs were heavily corrected for multiple comparisons. While this ensures the results reported here are robust, subtle effects may have been mitigated, which may explain why the amygdala was not detected in our functional connectivity networks (Wen et al., 2022) or why stimulus specificity was not detected during threat extinction despite other recent results showing stimulus-specific connectivity increases at the end of extinction (Wen et al., 2021).

In summary, using data-driven methods, we have characterized several functional connectivity networks involved in the Pavlovian threat learning paradigm. These functional connectivity networks overlap most commonly with areas involved in the default mode and salience networks, highlighting the potential importance of these canonical networks in threat acquisition, extinction, and extinction recall processes. This supports other recent findings from whole-brain connectivity analyses of fear conditioning that demonstrate the need for researchers to move beyond the previously focal region of interest analyses of fear conditioning paradigms in order to understand the dynamic nature of the human brain’s response in threat learning (Wen et al., 2021). The present work has the added benefit of refining these whole-brain functional connectivity patterns into spatially independent networks to identify which regions work directly in concert as well as which functional connectivity networks are involved in different aspects of threat learning and extinction. Future work will need to disentangle which of these networks are involved in automatic defensive responses to threat and which contribute to the actual subjective experience of fear and threat to better tailor clinical interventions for fear-related disorders to meet individual needs or symptom profiles.

## Supplementary Material

Supplementary Material

## Data Availability

All data produced in the present study are available upon reasonable request to the authors.
