# Supplementary material for: Broadening the scope: Multiple functional connectivity networks underlying threat conditioning and extinction
Source: Imaging Neurosci (Camb). 2024 Jul 3;2:imag-2-00213. doi: 10.1162/imag_a_00213 (PMC12272181; doi:10.1162/imag_a_00213)
Supplement: Supplementary Material [file imag_a_00213-supp.pdf]

Supplemental Information

Broadening the scope: Multiple functional connectivity networks underlying threat conditioning and extinction

Cody A. Cushing, Yujia Peng, Zachary Anderson, Katherine S. Young, Susan Y. Bookheimer, Richard E. Zinbarg, Robin Nusslock, Michelle G. Craske

Supplemental Methods

Skin conductance response (SCR) recording and preprocessing

Skin conductance response (SCR) data were recorded on a GSR100c amplifier and digitized using AcqKnowledge (Biopac Systems Inc., USA) from electrodes placed distally on the left index and middle fingers for the duration of each task phase. SCR data were sampled at 1kHz with 5 µS/V gain and preprocessed using ANSLAB (1). Data were visually inspected for movement artifacts that were removed on a trial-by-trial basis. A total of 54 participants were excluded for either remaining excessive motion artifacts after this step, technical issues during data collection, or a lack of variance in recording signal, resulting in 218 participants with analyzed SCR data across all task phases. This resulted in 180 participants with both SCR data and fMRI data during acquisition, 163 during extinction, and 144 during extinction recall. Stimulus-specific SCR responses were calculated by subtracting a 2 second baseline from the maximum value occurring between 0 to 6 seconds following stimulus onset. Data were normalized via a natural logarithm of 2+SCR transformation.

Site Differences in Spatial Maps

As our group ICA combined fMRI data from 2 different scanners, cross-site consistency in functional connectivity networks was assessed by testing IC spatial maps for effects of site. Specifically, participant-specific spatial maps for each IC network were submitted to an F-test in FSL's *randomise* (2) to test for any differences between the 2 sites. Non-parametric tests in *randomise* were performed using Threshold-Free Cluster Enhancement (TFCE) and 5000 permutations to correct for multiple comparisons within each test. Resulting corrected p-value maps were Bonferroni corrected for multiple comparisons across networks to match our main statistical analysis.

Generalizability of Results

As a test of the generalizability or replicability of any results found with this larger sample size, we performed a split-site cross-validation of our significant findings. Data were split by site and subjected to the same tests as described in *Statistical Analysis* with participant being the sole random effect variable.

Supplemental Results

CS-US contingency awareness

Participant CS-US contingency ratings collected at the end of teach task phase demonstrated a clear pattern of learning (Supplemental Figure 1). At the end of acquisition, participants rated CS-US contingency significantly higher for CS+ stimuli compared to the CS- ($t(222)=22.43$, $p<0.001$). At the end of extinction, there did still remain a significantly greater contingency rating for the CS+E compared to CS- ($t(207)=3.60$, $p<0.001$). However, there was strong evidence of extinction as contingency ratings significantly reduced for the CS+E at the end of extinction compared to the end of acquisition ($t(207)=-22.77$, $p<0.001$). Additionally, the difference in contingency ratings at the end of extinction (CS+ - CS-) was significantly and considerably smaller than the difference in contingency ratings at the end of acquisition ($t(207)=15.77$, $p<0.001$). At the end of extinction recall, there was no longer a significant difference in contingency ratings for the CS+E compared to the CS- ($t(204)=1.57$, $p=0.12$). There was a significant difference between the CS+U and the CS- ($t(204)=2.43$, $p=0.016$), but no significant difference between the CS+U and CS+E ($t(204)=0.58$, $p=0.56$).

SCR

Participant SCR responses collected throughout the task provided an additional measure of threat learning and extinction. In acquisition, SCR was greater for the CS+ compared to CS- ($t(181)=4.79$, $p<0.001$). In the last 4 trials of extinction, there was no difference in SCR for the CS+E compared to the CS- ($t(164)=0.39$, $p=0.70$). In the first 4 trials of extinction recall, SCR was significantly greater for the CS+U compared to CS+E ($t(143)=3.29$, $p=0.0013$). In the last 4 trials of extinction, this difference was no

longer significant ($t(143)=0.77$, $p=0.44$) as the CS+U goes through its own effective extinction during the extinction recall phase.

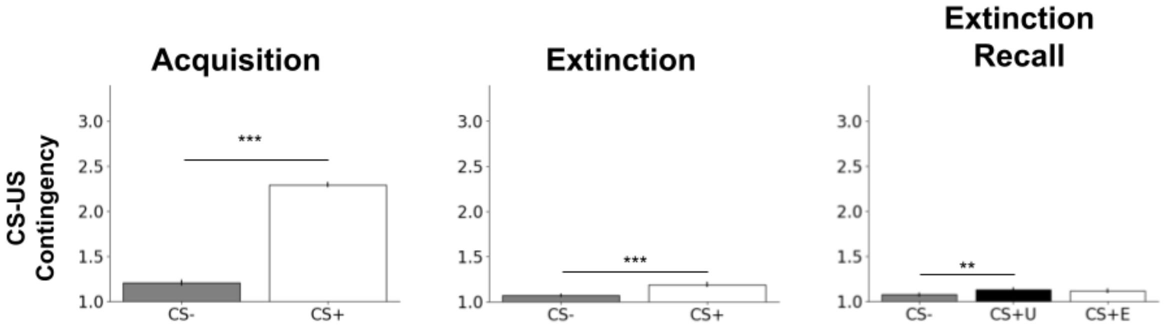

Supplemental Figure 1. Participant CS-US contingency ratings. At the end of each task phase (acquisition, extinction, and extinction recall), participants rated the CS-US contingency for each CS on a 3-point scale. Higher contingency ratings indicate a higher expectation of receiving a shock if they were to see that CS again. *** $p<0.001$, ** $p<0.01$

Association of effects with CS-US contingency awareness and SCR

During acquisition, the interaction effect reported in Fig. 1A was significantly correlated with CS-US contingency awareness ratings given at the end of the acquisition phase. Specifically, the difference in activity to CS+ and CS- in this functional connectivity network in late trials was correlated with the difference in contingency awareness ratings for the CS+ and CS- ($r(221)=-0.20$, $p=0.002$) such that the more negative CS+ was compared to CS-, the greater threat learning occurred (higher contingency for CS+>CS-). Also, the condition effect reported in the functional connectivity network in Fig. 2A was significantly correlated with contingency awareness ratings ($r(221)=0.26$, $p<0.001$) such that the higher the activity was for CS+ compared to CS-, the greater the threat learning. A similar correlation was found for the effect in Fig. 3B ($r(221)=0.17$, $p=0.01$) such that the greater the activity for CS+ compared to CS-,

the greater threat learning was observed. However, the effect in Fig. 3A did not correlate with contingency awareness ratings ($r(221)=0.0025$, $p=0.97$).

Despite these robust associations between our neural effects and behavioral measures of learning during acquisition, none of the observed effects were found to correlate with SCR (all p's>0.05). SCR did demonstrate significantly greater activity for the CS+ compared to CS- during acquisition ($t(222)=4.72$, $p<0.001$). However, there was also no correlation between SCR and behavioral contingency ratings ($r(178)=-0.024$, $p=0.74$)

There were no significant correlations between our observed neural effects and behavioral contingency ratings or SCR during extinction. This is perhaps unsurprising as contingency awareness ratings are only collected at the end of the task phase after extinction has already occurred and no differences between CS+ and CS- are expected.

As we were only experimentally interested in the early portion of extinction recall, no contingency ratings were available to test their correlation with our reported effect. Contingency ratings were collected at the end of the recall period after the CS+U had undergone its own effective extinction. We did observe a significant correlation between SCR and the neural effect reported in Fig. 2B ($r(142)=-0.20$, $p=.018$) such that the more negative the neural response to CS+U compared to CS+E, the greater the SCR response was in early trials to CS+U to CS+E.

In summary, effects during acquisition were robustly associated with participant behavioral contingency awareness ratings. The same correlation was not found during extinction but the task was not designed to optimally capture relations between neural responding and behavioral responding. There was a significant correlation between the SCR response during extinction recall and our observed functional connectivity network activity. However, the other findings did not correlate with SCR well. This is likely

attributable to the noisy and tenuous nature of SCR as SCR response similarly did not correlate with behavioral responses despite both measures showing robust effects of threat learning and extinction.

Relation of participant symptoms to reported effects

Although the goal of the present study was to characterize large-scale functional connectivity networks involved in threat learning and extinction regardless of participant symptom levels, we explored the potential impact of participant symptoms on the reported effects. Each effect was correlated to our 3 dimensional measures of participant symptoms: General Distress, Fears, and Anhedonia-Apprehension. By and large, these symptoms had no relation to the reported effects. There was some preliminary evidence that Anhedonia-Apprehension was associated with the effect reported in Fig. 1A ($r(222)=0.14$, $p=0.032$ uncorrected). This association indicates that participants with higher levels of anhedonia had greater difference in CS- vs CS+ responding in late acquisition in this particular functional connectivity network (Fig. 1A). However, this association would not survive any correction for multiple comparisons.

Potential contamination of US response during CS

While we took precautions in our statistical analysis to minimize the impact of US responding on CS responses, there is always the concern that responses to the US may contaminate CS responses during the acquisition phase in an fMRI study due to the sluggish and temporally delayed nature of the BOLD signal. We did not choose to analyze only unreinforced CS trials during our main analysis as our reinforcement rate (62.5%) would have resulted in a substantial reduction of power. As an auxiliary test of US response contamination during acquisition, we analyzed the interaction effect from Fig. 1A using unreinforced CS trials. Despite the reduced power (1-2 trials each in the early vs. late blocks), the interaction was still highly significant ($F(1,885)=20.28$, $p<0.001$ Bonferroni corrected). For the main effects of condition reported in Fig. 2A and Fig. 3, we modeled all unreinforced CS+ trials from

acquisition (rather than just the early vs. late trials) for increased power due to these effects not being time sensitive. Each of these effects was similarly still highly significant (all *p*'s<0.001 Bonferroni corrected) when only analyzing unreinforced CS trials. Collectively, this indicates that none of our observed effects during acquisition are due to contamination from the US response.

Cross-site consistency of functional connectivity

To ensure our reported functional connectivity networks were representative of both study sites (UCLA and NU), we tested each independent component for effects of study site. One functional network (reported in Fig. 3B) did demonstrate a small effect of site in 2 clusters (cluster 1 extent: 490 voxels; cluster 2 extent: 481 voxels) near the parietal operculum cortex. However, follow-up FSL randomise tests on data from each site revealed significant connectivity for each site individually through the entirety of these clusters. That is, despite the appearance of a site effect in this region, both sites still had significant connectivity in this area on their own. No other networks had any significant site differences. In sum, all of our reported networks are representative of both study sites with no meaningful differences between sites in our multi-site group ICA.

Cross-site generalization of effects

As a preliminary test of how generalizable and replicable the reported results may be, we also performed a split-site validation of our primary findings. Full results for each study site are reported in Table 1. All findings are independently significant at both sites, with the exception of one effect (reported in Fig. 4B) that did not quite reach significance at the UCLA site independently (*p*=0.11). This is likely due to an effective halving of our statistical power when splitting by site. In summary, our reported effects are broadly generalizable across both our study sites, indicating a high chance of replication in independent datasets.

Supplemental Table 1. Results for each study site independently.

**Reported Effect**	**UCLA**<br><br>Uncorrected p-value	**NU**<br><br>Uncorrected p-value
Fig. 1A	0.0035	0.0056
Fig. 1B	0.028	0.01
Fig. 2A	0.027	0.00063
Fig. 2B	0.0067	0.049
Fig. 3A	0.0013	0.042
Fig. 3B	2.2e-11	1.7e-16
Fig. 4A	0.031	5.0e-8
Fig. 4B	0.11	5.5e-6

References

1. Blechert J, Peyk P, Liedlgruber M, Wilhelm FH. ANSLAB: Integrated multichannel peripheral biosignal processing in psychophysiological science. Behav Res. 2016 Dec 1;48(4):1528–45.

2. Winkler AM, Ridgway GR, Webster MA, Smith SM, Nichols TE. Permutation inference for the general linear model. NeuroImage. 2014 May 15;92:381–97.
